# Quality Measurement of Two-dimensional Shear Wave Speed Imaging for Breast Lesions: the Associated Factors and the Impact to Diagnostic Performance

**DOI:** 10.1038/s41598-017-05281-5

**Published:** 2017-07-11

**Authors:** Dan-Dan Li, Hui-Xiong Xu, Bo-Ji Liu, Xiao-Wan Bo, Xiao-Long Li, Rong Wu

**Affiliations:** Department of Medical Ultrasound, Shanghai Tenth People’s Hospital, Ultrasound Research and Education Institute, Tongji University School of Medicine, Shanghai, 200072 China

## Abstract

This study aimed to identify the associated factors for quality measurement (QM) of shear wave speed (SWS) imaging and to validate the additional value of QM in the diagnosis of breast lesions. From September 2014 to February 2015, conventional ultrasound and SWS imaging were performed in 338 women with 361 breast lesions. Binary logistic regression was used to identify associated factors for QM. Sensitivity, specificity and the area under receiver operating characteristic (ROC) curve (AUC) among maximum SWS (SWS_*max*_), QM and SWS_*max*_ plus QM (SWS_*max*_+QM) were compared to validate additional value of QM. Pathology confirmed 263 (72.9%) benign lesions and 98 (27.1%) malignancies. Maximum depth (Odds ratio [OR]: 1.398) and posterior features (OR: 1.206) were identified as independent associated factors for QM. Compared with SWS_*max*_ and QM, the sensitivity of SWS_*max*_+QM increased from 67.3%, 64.3% to 83.7% whereas the specificity decreased from 90.5%, 72.6% to 65.4% (all *P* < 0.05). SWS_*max*_ had the highest AUC in comparison with QM and SWS_*max*_+QM (0.849 vs. 0.685 vs. 0.745; *P* < 0.05). QM for breast lesions is associated with maximum depth and posterior features. Adding QM to SWS_*max*_ is useful for breast cancer screening and SWS_*max*_ alone is useful for breast cancer differentiation.

## Introduction

Shear wave elastography (SWE) is a useful adjunct to conventional ultrasound (US) in the diagnosis of breast lesions, which provides quantitative information of tissue stiffness and improves diagnostic performance^1^. Two kinds of SWE including point shear wave speed (P-SWS) measurement and shear wave speed (SWS) imaging are usually used in breast^2^. P-SWS measurement such as Virtual Touch Tissue Quantification (VTQ; Siemens Medical Solutions, Mountain View, CA, USA) has been proven to have a good diagnostic performance in the diagnosis of breast lesions, whereas invalid SWS measurements are often encountered and displayed as X.XX m/s, which leads to uncertainty of the true stiffness of the targeted area^3–7^. Recently, it is realized that the invalid SWS measurement might be caused by low quality of shear wave (SW) propagation in the targeted tissue. The shear wave (SW) quality may be affected by the motion of transducer or patients, lesion depth, tissue inhomogeneity, intralesional calcifications, and others^8, 9^, which leads to the SW in the lesion has substantial noise or minimal tissue displacement and is not accurately interpretable.

Technical innovations have made display and measurement of the SW quality possible. One of them is to provide a two-dimensional (2D) quality measurement (QM) map, as it does with a recently developed SWS imaging technique (i.e. Virtual Touch Tissue Imaging and Quantification, VTIQ; Siemens Medical Solutions, Mountain View, CA, USA), in which the quality of SW propagation is displayed in different 2D colors^8^. QM is an important issue when applying SWS imaging to evaluate breast lesions. With the 2D QM map as a reference, the areas of low QM could be avoided easily when performing SWS measurement, which would reduce the invalid stiffness measurements and lead to a more accurate cut-off value for diagnosing breast lesions. Hence, compared with using P-SWS measurement, the reliability of SWS measured by SWS imaging is guaranteed with the help of QM map.

In addition to be used as a reference for selecting shear wave region of interest (SW-ROI) for SWS measurement, Barr *et al*. recently found another use of QM in the diagnosis of breast lesions^9^. They found the combination of QM and SWS measurement improved the sensitivity from 50% to 93% without significant change in specificity (from 94% to 89%) and they believed that low QM might be a feature of malignancy^9^. However, low QM of SWS imaging is also encountered in benign breast lesions, especially in benign ones with large volume^8, 9^. Therefore, it is important to find out the associated factors for QM of SWS imaging, which not only helps operators to avoid the possible factors under specific conditions, but also helps the operators to evaluate the SWS measurement results more objectively. In the present study, it was aimed to identify the possible associated factors for QM in breast lesions and to validate the additional value of QM in the diagnosis of breast lesions.

## Results

**Figure 1:**
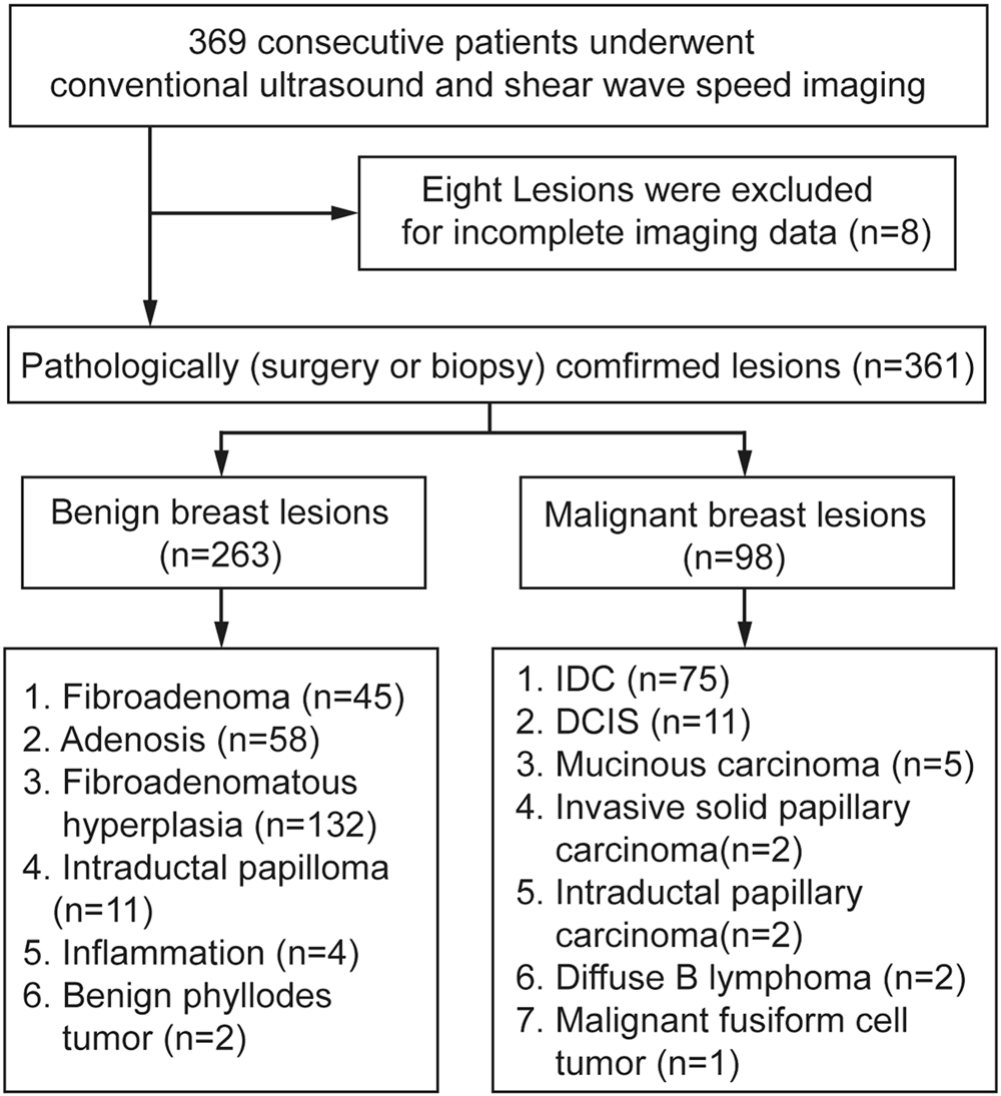
Flowchart of breast lesions selection. IDC = Infiltrating ductal carcinoma; DCIS = Ductal carcinoma in situ.

The pathology confirmed 263 (72.9%, 263/361) benign lesions and 98 (27.1%, 98/361) malignant lesions (Fig. 1). The mean age of patients with malignant breast lesions was significantly higher than those with benign breast lesions (57.0 years ± 12.2vs. 40.0 years ± 12.8) (*P* < 0.05) and the maximum depth of malignant breast lesions was significantly higher than that of the benign ones (21.2 mm ± 7.1 vs. 15.4 mm ± 4.7) (*P* < 0.05), which were also found in three subgroups with different diameters (all *P* < 0.05) (Table 1). In terms of conventional US features of breast lesions, irregular shape, non-parallel orientation, non-circumscribed margin, changed posterior features, calcification and vascularity were more often found in malignant breast lesions (all *P* < 0.05) (Table 1). In Group 1, there were no significant differences between benign and malignant breast lesions in posterior features, calcification and vascularity (all *P* > 0.05); however, significant differences were found in Group 2 (all *P* < 0.05). In Group 3, no significant difference was found in vascularity between benign and malignant breast lesions (Table 2).

**Table 1:** The Characteristics of Patients, US and SWS Imaging Features of Breast Lesions.

Characteristic	Overall	Malignant	Benign	*P*-value
Patients	338	98 (29.0%)	240 (71.0%)	
Mean age (yrs)	45.0 ± 14.8 (16–84)	57.0 ± 12.2 (26–84)	40.0 ± 12.8 (16–80)	<0.001*
Lesions	361	98 (27.1%)	263 (72.9%)	
Lesion position				0.993
Right	173 (47.9%)	47 (48.0%)	126 (47.9%)	
Left	188 (52.1%)	51 (52.0%)	137 (52.1%)	
Diameter (mm)	17.0 ± 10.6 (4–86)	22.3 ± 13.7 (6–86)	15.0 ± 8.4 (4–71)	<0.001*
Maximum depth (mm)	17.0 ± 6.1 (7–43)	21.2 ± 7.1 (8–43)	15.4 ± 4.7 (7–31)	<0.001*
Shape				<0.001*
Oval/Round	176 (48.7%)	9 (9.2%)	167 (63.5%)	
Irregular	185 (51.2%)	89 (90.8%)	96 (36.5%)	
Orientation				<0.001*
Parallel	235 (65.1%)	32 (32.7%)	203 (77.2%)	
Not parallel	126 (34.9%)	66 (67.3%)	60 (22.8%)	
Margin				<0.001*
Circumscribed	178 (49.3%)	8 (8.2%)	170 (64.6%)	
Non-circumscribed	183 (50.7%)	90 (91.8%)	93 (35.4%)	
Echo pattern				0.415
Isoechoic	1 (0.3%)	0 (0%)	1 (0.4%)	
Complex cystic	9 (2.5%)	4 (4.1%)	5 (1.9%)	
Hypoechoic	351 (97.2%)	94 (95.9%)	257 (97.7%)	
Posterior features				<0.001*
Changed	109 (30.2%)	55 (56.1%)	54 (20.5%)	
Unchanged	252 (69.8%)	43 (43.9%)	209 (79.5%)	
Calcifications				<0.001*
Present	70 (19.4%)	37 (37.8%)	33 (12.5%)	
Absent	291 (80.6%)	61 (62.2%)	230 (87.5%)	
Vascularity				<0.001*
Present	197 (54.6%)	84 (85.7%)	113 (43.0%)	
Absent	164 (45.4%)	14 (14.3%)	150 (57.0%)	
Maximum SWS (m/s)	4.59 ± 2.12 (1.45–10)	6.70 ± 2.27 (2.84–10)	3.80 ± 1.41 (1.45–9.75)	<0.001*

*Indicates a significant difference.

US = ultrasound; SWS = shear wave speed; Changed posterior features include enhancement, shadowing and combined pattern.

**Table 2:** The characteristics of patients and the US features of breast lesions in subgroups.

Characteristic	Group 1, lesions ≤10 mm (n = 91)		p-value	Group 2, lesions 11–20 mm (n = 184)		p-value	Group 3, lesions > 20 mm (n = 86)		p-value
	Malignant	Benign		Malignant	Benign		Malignant	Benign	
**Patients (n)**	12 (15.2%)	67 (84.8%)		42 (23.7%)	135 (76.3%)		44 (53.7%)	38 (46.3%)	
**Mean age (yrs)**	60.1 ± 9.1 (45–79)	45.5 ± 12.1 (22–75)	<0.001*	58.6 ± 12.1 (37−84)	38.9 ± 11.8 (20−79)	<0.001*	54.5 ± 13.2 (26−82)	33.5 ± 14.0 (16−80)	<0.001*
**Lesions (n)**	12 (13.2%)	79 (86.8%)		42 (22.8%)	142 (77.2%)		44 (51.2%)	42 (48.8%)	
**Lesion position (n)**			0.563			0.748			0.817
**Right**	5 (41.7%)	40 (50.6%)		21 (50.0%)	67 (47.2%)		21 (47.7%)	19 (45.2%)	
**Left**	7 (58.3%)	39 (49.4%)		21 (40.0%)	75 (52.8%)		23 (52.3%)	23 (54.7%)	
**Diameter (mm)**	8.6 ± 1.5 (6−10)	8.2 ± 1.6 (4−10)	0.405	15.1 ± 3.1 (11−20)	14.6 ± 2.8 (11−20)	0.352	32.9 ± 13.9 (21−86)	29.4 ± 10.6 (21−71)	0.195
**Maximum depth (mm)**	16.3 ± 4.6 (10−27)	13.7 ± 3.7 (8−23)	0.037*	19.3 ± 5.3 (8−30)	15.3 ± 4.6 (7−30)	<0.001*	24.6 ± 7.6 (11−43)	19.0 ± 5.2 (11−31)	<0.001*
**Shape (n)**			0.003*			<0.001*			<0.001*
**Oval/Round**	3 (25.0%)	58 (73.4%)		6 (14.3%)	86 (60.6%)		0 (0%)	24 (57.1%)	
**Irregular**	9 (75.0%)	21 (26.6%)		36 (85.7%)	56 (39.4%)		44 (100%)	18 (42.9%)	
**Orientation (n)**			0.002*			<0.001*			0.001*
**Parallel**	2 (16.7%)	54 (68.4%)		11 (26.2%)	116 (81.7%)		19 (43.2%)	33 (78.6%)	
**Not parallel**	10 (83.3%)	25 (31.6%)		31 (73.8%)	26 (18.3%)		25 (56.8%)	9 (21.4%)	
**Margin (n)**			0.010*			<0.001*			<0.001*
**Circumscribed**	3 (25.0%)	54 (68.4%)		4 (9.5%)	91 (64.1%)		2 (4.5%)	26 (61.9%)	
**Non-circumscribed**	9 (75.0%)	25 (31.6%)		38 (90.5%)	51 (35.9%)		42 (95.5%)	16 (38.1%)	
**Echo pattern (n)**			—			—			—
**Isoechoic**	0 (0%)	0 (0%)		0 (0%)	1 (0.7%)		0 (0%)	0 (0%)	
**Complex cystic**	0 (0%)	0 (0%)		0 (0%)	2 (1.4%)		4 (9.1%)	3 (7.1%)	
**Hypoechoic**	12 (100%)	79 (100%)		42 (100%)	139 (97.9%)		40 (90.9%)	39 (92.9%)	
**Posterior features (n)**			0.128			<0.001*			0.018*
**Changed**	5 (41.7%)	14 (17.7%)		22 (52.4%)	24 (16.9%)		28 (63.6%)	16 (38.1%)	
**Unchanged**	7 (58.3%)	65 (82.3%)		20 (47.6%)	118 (83.1%)		16 (36.4%)	26 (61.9%)	
**Calcifications (n)**			1.000			0.002*			<0.001*
**Present**	2 (16.7%)	10 (12.7%)		13 (31.0%)	16 (11.3%)		22 (50.0%)	5 (11.9%)	
**Absent**	10 (83.3%)	69 (87.3%)		29 (69.0%)	126 (88.7%)		22 (50.0%)	37 (88.1%)	
**Vascularity (n)**			0.060			<0.001*			0.078
**Present**	6 (50.0%)	16 (20.3%)		36 (85.7%)	63 (44.4%)		42 (95.5%)	34 (81.0%)	
**Absent**	6 (50.0%)	63 (79.7%)		6 (14.3%)	79 (55.6%)		2 (4.5%)	8 (19.0%)	
**Maximum SWV (n)**	5.05 ± 2.11 (2.84−9.43)	3.58 ± 1.45 (1.45−9.75)	0.003*	6.26 ± 2.35 (3.09−10)	3.89 ± 1.43 (1.71−8.99)	<0.001*	7.58 ± 1.85 (3.33−10)	3.95 ± 1.18 (2.16−7.92)	<0.001*

*Indicates a significant difference.

US = ultrasound

SWV = shear wave velocity

Changed posterior features included enhancement, shadowing and combined pattern.

### Factors associated with QM

Binary logistic regression analysis showed that maximum depth of breast lesions was the independent associated factor for low QM with an odds ratio (OR) of 1.398 (95% CI: 1.296–1.509) in all breast lesions, followed by posterior features on conventional US (OR: 1.206; 95% CI: 1.044–1.394). In Group 1, lesion margin was an independent associated factor (OR: 5.533; 95% CI: 1.166–26.260), followed by maximum lesion depth (OR: 1.505; 95% CI: 1.217–1.861). In Group 2, calcification (OR: 6.947; 95% CI: 2.113–22.847) and maximum lesion depth (OR: 1.633; 95% CI: 1.421–1.876) were the independent associated factors; While in Group 3, only the lesion depth was identified as the independent associated factor with an OR of 1.218 (95% CI: 1.103–1.345) (Table 3). There were 56 of 72 benign breast lesions with low QM appeared in the periphery of the lesion. The results of the associated factors for QM by the two independent observers are shown in Appendix 1.

**Table 3:** Binary Logistic Regression Analysis in the Prediction of Low Quality Measurement for Breast Lesions.

Factors	OR	95% CI	*P*-Value
Overall (n = 361)			
Maximum depth	1.398	1.296, 1.509	<0.001
Posterior features	1.206	1.044, 1.394	0.011
≤10 mm Lesions (n = 91)			
Maximum depth	1.505	1.217, 1.861	<0.001
Margin	5.533	1.166, 26.260	0.031
11–20 mm Lesions (n = 184)			
Maximum depth	1.633	1.421, 1.876	<0.001
Calcifications	6.947	2.113, 22.847	0.001
>20 mm Lesions (n = 86)			
Maximum depth	1.218	1.103, 1.345	<0.001

OR = Odds ratio;

Posterior features = enhancement, shadowing and combined pattern.

### Diagnostic performance of SWS imaging

**Figure 2:**
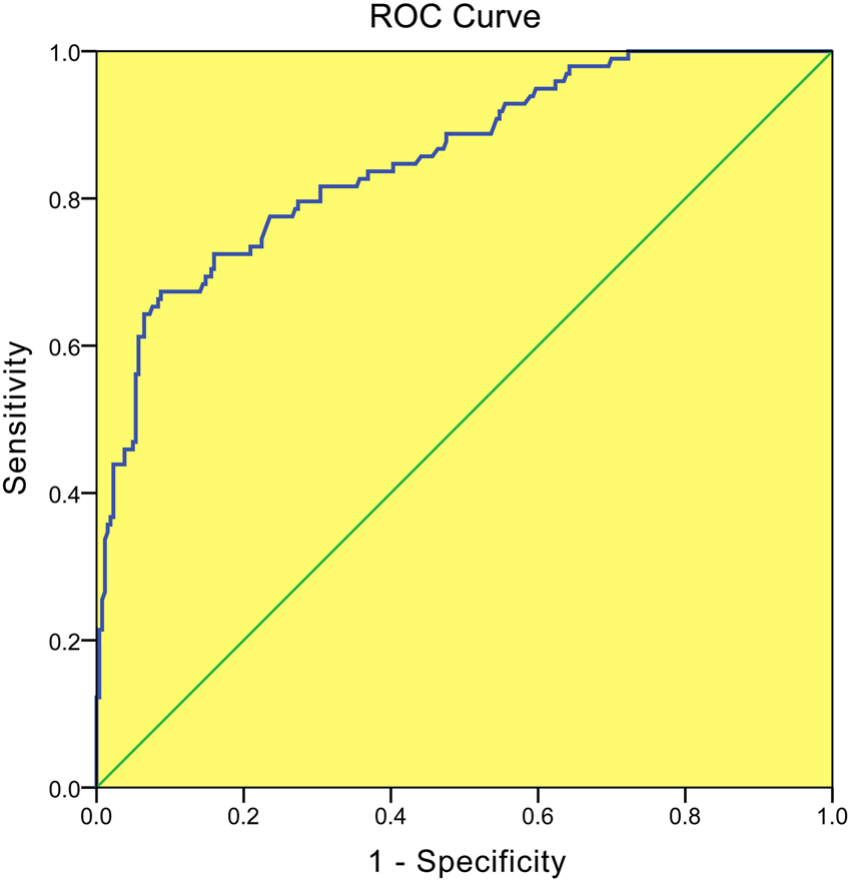
The operating characteristic (ROC) curve of SWS_max_.

The maximum SWS (SWS_*max*_) of malignant breast lesions (6.70 m/s ± 2.27) was significant higher than that of benign breast lesions (3.80 m/s ± 1.41) (*P* < 0.05). There were 91 breast lesions with SWS_*max*_ ≥ 5.80 m/s, which was the cut-off value of SWS_*max*_, and 270 breast lesions with SWS_*max*_ < 5.80 m/s (Fig. 2). By the method of SWS_*max*_, 57 breast lesions were misdiagnosed, including 32 false negative results and 25 false positive results. SWS_*max*_ alone had a high specificity (90.5%, 238/263) whereas the sensitivity was only 67.3% (66/98).

### The impact of QM to Diagnostic Performance

**Figure 3:**
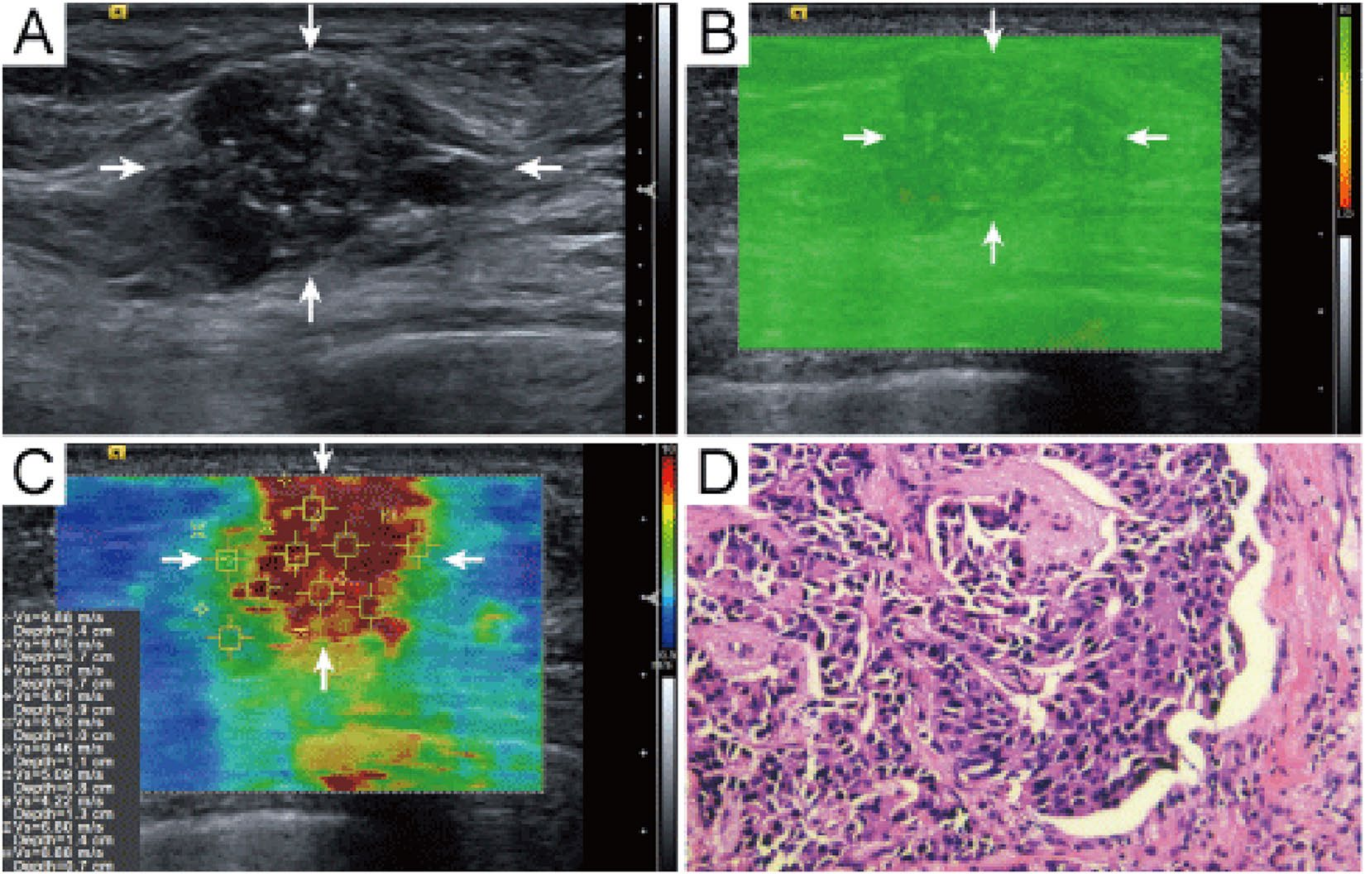
Invasive ductal carcinoma in a 70 years old woman. (**A**) The lesion (arrows) is classified as US BI-RADS category 5 on B-mode US. (**B**) The whole lesion (arrows) is covered by green color on QM mode of SWS imaging, indicating high QM, although calcifications are observed. (**C**) The SWSmax of the lesion (arrows) is 9.88 m/sec. (**D**) Pathological examination confirms the diagnosis of invasive ductal carcinoma (hematoxylin-eosin stain; original magnification, 200). QM = quality measurement.

High QM was found in 226 (62.6%, 226/361) breast lesions while low QM was found in 135 (37.4%, 135/361) breast lesions. In benign breast lesions, 191 out of 263 (72.6%) lesions had high QM (Fig. 3), while in malignant breast lesions 63 out of 98 (64.3%) lesions had low QM (Fig. 2) (P < 0.05). By the method of QM, there were 21 out of 35 (60.0%) false negative results with depth <17 mm and 22 out of 35 (62.9%) false negative results without changed posterior features, while there were 57 out of 72 (79.2%) false positive results with depth ≥17 mm and 29 out of 72 (40.2%) false positive results with changed posterior features. By SWS_*max*_ alone, there were 12 out of 32 (37.5%) false negative results with depth <17 mm and 16 out of 32 (50.0%) false negative results without changed posterior features, while there were 3 out of 24 (12.5%) false positive results with depth ≥17 mm and 5 out of 24 (20.8%) false positive results with changed posterior features. After using additional method of QM to SWS_*max*_, additional 82 out of 270 SWS_*max*_-benign breast lesions were classified into the malignant group, of which 66 (80.5%, 66/82) lesions were misdiagnosed.

Compared with SWS_*max*_ alone and QM alone, the sensitivity of SWS_*max*_+QM increased from 67.3%, 64.3% to 83.7% (82/98) whereas the specificity decreased from 90.5%, 72.6% to 65.4% (172/263) (all *P* < 0.05). The method of SWS_*max*_ alone (AUC: 0.849; 95% CI: 0.808–0.885) had the highest diagnostic performance in comparison with QM alone (AUC: 0.685; 95% CI: 0.634–0.732) and SWS_*max*_+QM (AUC: 0.745; 95% CI: 0.697–0.790) (all *P* < 0.05) (Table 4). The diagnostic performances of SWS_*max*_+QM method for the two independent observers are shown in Appendix 2.

**Table 4:** The Diagnostic Performances of all the Method.

Group	Cut-off	SEN (95%CI)	*P*-Value	SPE (95%CI)	*P*-Value	NPV (95%CI)	PPV (95%CI)	AUC	*P*-Value
QM alone	low	64.3% (54.0–73.7%)	0.736^**$**^	72.6% (66.8–77.9%)	<0.001^**$**^	46.7% (38.0–55.4%)	84.5% (79.1–89.0%)	0.685 (0.634–0.732)	<0.001^**$**^
Group 1 (n = 91)	low	25.0% (5.5–57.2%)		87.3% (78.0–93.8%)		88.5% (79.2–94.6%)	23.1% (5.0–53.8%)	0.562 (0.454–0.666)	0.050
Group 2 (n = 184)	low	66.7% (50.5–80.4%)		71.1% (62.9–78.4%)		87.8% (80.4%-93.2%)	40.6% (28.8–53.2%)	0.689 (0.617–0.755)	0.038^**$**^
Group 3 (n = 86)	low	72.7% (57.2–85.0%)		50.0% (34.2–65.8%)		63.6% (45.1–79.6%)	60.4% (46.0–73.5%)	0.614 (0.502–0.717)	<001^**$**^
SWS_max_ alone	5.80 m/s	67.3%* (57.1–76.5%)	<0.001*	90.5%* (86.7–94.1%)	<0.001*	88.1% (63.0–82.1%)	72.5% (83.7–91.8%)	0.849 (0.808–0.885)	<0.001*
Group 1 (n = 91)	3.97 m/s	66.7% (34.9–90.1%)		72.2% (60.9–81.7%)		26.7% (12.3–45.9%)	93.4% (84.1–98.2%)	0.743 (0.641–0.829)	0.166
Group 2 (n = 184)	6.19 m/s	47.62 (32.0–63.6%)		95.5% (90.9–98.2%)		74.1% (53.3–89.1%)	87.0% (81.0–91.7%)	0.783 (0.719–0.839)	0.037*
Group 3 (n = 86)	4.99 m/s	90.9% (78.3–97.5%)		92.9% (80.5–98.5%)		93% (80.7–98.6%)	90.7% (77.9–97.4%)	0.935 (0.861–0.977)	<0.001*
SWS_max_ + QM	—	83.7% (74.8–90.4%)	<0.001^#^	65.4% (59.3–71.1%)	<0.001^#^	91.5% (39.8–55.1%)	47.4% (86.5–95.1%)	0.745 (0.697–0.790)	0.005^#^
Group1 (n = 91)	—	50.0% (21.1–78.9%)		79.8% (69.2–88.0%)		27.3% (10.4–50.8)	91.3% (82.0–96.7%)	0.649 (0.542–0.746)	0.194
Group2 (n = 184)	—	73.8% (58.0%-86.1%)		65.6% (57.5–73.0%)		36.9% (26.6–48.2%)	90.2% (83.1–95.0)	0.697 (0.627–0.760)	0.326
Group3 (n = 86)	—	95.5% (84.5–99.4%)		47.6% (32.0–63.6%)		65.6% (52.7–77.1%)	90.9% (70.2–99.0%)	0.715 (0.608–0.808)	0.003^#^

Group1, ≤10 mm; Group 2, 11–20 mm; Group 3, >20 mm.

95%CI = 95% confidence interval.

AUC = the area under the curve;

QM = quality measurement;

SWS_max_ = maximum shear wave speed;

NPV = negative predictive value;

PPV = positive predictive value.

SEN = sensitivity;

SPE = specificity;

*Indicates a significant difference SWS_max_ vs. SWS_max_+QM.

^#^Indicate a significant difference QM vs. SWS_max_+QM.

^$^Indicate a significant difference QM vs. SWS_max_.

The intra-operator and inter-operator consistency for SWS_*max*_ measurement were excellent with an overall ICC of 0.910 (95% CI: 0.827–0.954) and 0.790 (95% CI: 0.620–0.889) respectively. In addition, the intra-operator and inter-operator consistency for QM were excellent with a Kappa value of 0.939 (95% CI: 0.795–1.000) and 0.816 (95% CI: 0.588–1.000) respectively.

## Discussion

QM is an important issue when applying SWS imaging to evaluate breast lesions. The SW-quality mode displays a 2D quality map, which is generated by estimation on each sample position including signal-to-noise ratio (SNR) in the acquired echo signals and SNR of the detected displacement data. The latter two are indicative of the quality of SW propagation. Measurement errors about the SW propagation may occur in the course of data acquisition and data processing. Patient and transducer motion may occur in the process of data acquisition, whereas low SNR signals may occur in data processing. After excluding the factors of patient or transducer motion, Barr *et al*. reported that an addition of a QM plus SWS measurement would further improve the accuracy of SWS imaging and help to eliminate false negative lesions^9^. They suggested that if a lesion is not color coded or has a poor QM and is not cystic, it has a high probability of being a malignancy. In 2015, the issue of QM was discussed in the World Federation of Ultrasound in Medicine and Biology (WFUMB) guideline and it was indicated that the quality of SWS imaging was associated with SW propagation and SW may not propagate in very stiff breast lesions^10^. Besides pre-compression, many studies found that low QM or invalid SWS measurement (unidentified SW or not color-coded SWS images) was more frequently observed in malignant breast lesions^8–14^. This phenomenon indicated that SW may not propagate as expected in some cancers^10^. On the other hand, invalid SWS measurements can also be found in solid or predominantly solid benign breast lesions other than cystic benign lesions. Therefore, low QM or invalid SWS measurement is not a unique feature for malignancy. Unfortunately, although invalid SWS measurements are often encountered in clinical practice when applying SWE, the underlying mechanism is still unknown, which causes a dilemma in dealing with this situation in clinical practice.

Regions of low QM on SW-quality image indicate where the SWS estimations may be less accurate and reliable for poor signal quality. In the present study, low QM was more frequently found in malignant lesions (64.3%, 63/98) than in benign lesions (27.4%, 72/263), which was consistent with the results of previous studies^9, 15^. However, the pathologic results of tumors were failed to be identified as an independent associated factor for QM by binary logistic regression analysis. On the other hand, maximum depth of breast lesion was revealed to be an independent associated factor for QM, with an overall OR of 1.4. In the subgroups, OR values of maximum depth for Group 1, Group 2 and Group 3 were 1.5, 1.6 and 1.2, respectively. In 135 breast lesions with low QM, 115 (85.2%) breast lesions had maximum depth of more than or equal to 17 mm, the mean maximum depth in the study. All these results indicated that maximum depth of breast lesion had substantial influence on QM, which was consistent with the results of Chang *et al*. for strain elastography^16–18^. Compared with using manual compression, SWS imaging could generate adequate force to achieve measurable displacement within the depth of greater than 4 to 4.5 cm using the acoustic radiation force impulse (ARFI)^9, 19^. However, the depth of lesion still would affect QM in superficial organ such as breast. This contradiction may be related to the assumption in imaging principle of SWE. The former conclusion is established under the assumption of medium homogeneity while in fact the breast tissue is far from homogeneous. On the other hand, the quality of SWS imaging was reported depended on the image size^20^. With the depth of the breast lesion increases, the size of SWS imaging image would be increased, which may explain the effect of breast lesion depth on QM. In addition, ARFI push pulse attenuation along with lesion depth might also be another important mechanism. Although the increase in ARFI output can compensate for SW attenuation and reduce the rate of low QM, its application to human tissues is limited due to the concern of safety^21–24^.

The other associated factor for QM was changed posterior features of breast lesion with an overall OR of 1.2. The changed posterior features of breast lesion include posterior acoustic enhancement, acoustic shadowing and their combination, which always indicate inhomogeneous tissue in breast lesions. The changed posterior features are associated with pathological changes such as cystic degeneration, focal hyaline degeneration, liquefaction necrosis, and calcifications. These changes lead to inhomogeneity of tissue, which in turn interfere or block the transverse propagation of SW in the targeted tissue. When the SW propagation is interrupted by a sudden decrease or a sudden increase of tissue stiffness, the SW would attenuate significantly and thus lead to a low QM. The two associated factors of QM had effect on the diagnostic performance of SWS imaging. For QM alone and SWS_*max*_ alone, there were 60% and 38% false negative results came from the breast lesions with depth <17 mm and 63% and 50% false negative lesions without changed posterior features respectively, while there were 79% and 13% false positive results came from breast lesions with depth ≥17 mm and 40% and 21% false positive lesions with changed posterior features. The depth and changed posterior features of breast lesion led more false negatives and false positive by QM than by SWS_*max*_, which indicated that the effect of depth and changed posterior features on the diagnostic performance of SWS was less than estimated although the depth and posterior features had great effect on the quality of shear wave.

In subgroup analysis, non-circumscribed margin and calcifications were found to be independent associated factors for QM in Group 1 and Group 2 respectively. Non-circumscribed margin and calcifications may be related to the active cell growth or relative dystrophia, which also cause inhomogeneous tissue and the resultant deformation or blockade of SW propagation^25^. However, these two factors were not applicable to all breast lesions. One explanation was possibly the lower rates of non-circumscribed margin or calcification in all breast lesions than in the subgroups, and the other was that QM might be associated with the degree of stiffness inhomogeneity.

Interestingly, low QM tended to appear in the periphery of benign breast lesion with several small yellow areas seen at the periphery of the lesion (56/72). It could be explained by the acoustic wave reflection. When transverse SW is incident on a smooth margin of benign breast lesions, such as fiber capsule of fibroadenoma, transverse propagation of SW is reflected back, leading to a low QM.

As to the technical factors having effect on the QM, it is essentially a value computed as a weighted combination of the echo SNR, displacement SNR, and normalized cross correlation coefficient between the two displaced waveforms^9^. A poor QM is with a value equal or more than 0.87^9^. In other words, if the echo signal and displacement signal that could be detected by the transducer decreased or there were too much noise signal leading to the value of QM less than 0.87, the QM would be poor.

In the study, the specificity of SWS_*max*_+QM decreased by 25% compared with SWS_*max*_ alone. In addition QM alone had moderate sensitivity and specificity in the diagnosis of breast lesions. It seemed that the usefulness of QM in the diagnosis of breast lesions was not so high as expected. Nevertheless, with the help of 2D QM map, the areas of low QM could be avoided easily to reduce invalid stiffness measurements when performing on-site SWS measurement. What’s more, the sensitivity of SWS imaging significantly increased after adding QM to SWS_*max*_, which indicated that QM could help reduce the false negative results of SWS imaging and reduce the misdiagnosis of malignant breast lesions. It is very important when doing screening for breast cancer. The earlier the breast cancer is detected, the better the prognosis will be for patients. Compared with breast cancer screening, it is better to choose SWS_*max*_ alone than to choose SWS_*max*_+QM when differentiating benign and malignant breast lesions. Not only the excellent specificity of SWS_*max*_ had been shown in the study, but also the significant decrease in specificity of SWS_*max*_ had been observed after combining the additional use of QM. In Group 3 (diameter >20 mm), the diagnostic performance of SWS_*max*_ was excellent with AUC of 0.935, sensitivity of 91% and specificity of 93%, while the diagnostic performances were moderate in other two groups (diameter ≤20 mm), indicating that SWS_*max*_ was particularly useful in the diagnosis of breast lesions with diameter greater than 20 mm.

There were several limitations in the study. Only patients and lesions associated factors were analyzed, while apparatus related factors, such as thermal noise, finite spatial and temporal resolution and finite signal bandwidth were not included into analysis^26^. Although the intra-operator consistency and the inter-operator consistency of QM were excellent with a Kappa value of 0.939 and 0.816 respectively, the effect of operator independence for QM still could not be neglected. Only one type of SWS imaging equipment was used in the study, and the results might have varied from different types of elastography equipment. Due to the retrospective feature of the study, selection bias could not be avoided, the results of the study should be validated in future prospective studies.

In conclusion, QM for breast lesions is associated with maximum depth and posterior features. Adding QM to SWS_*max*_ is useful for breast cancer screening and SWS_*max*_ alone is useful for breast cancer differentiation.

## Materials and Methods

### Patients

The Ethics Committee of Shanghai Tenth People’s Hospital approved this retrospective study and informed consents of all patients were obtained. All the methods involved in this study were performed in accordance with the relevant guidelines and regulations. From September 2014 to February 2015, 731 consecutive patients with breast lesions palpable by clinicians or with an US-detected breast lesion were scheduled to conventional US examination and SWS imaging examination. The inclusion criteria were as following: (1) breast lesions were able to be detected by conventional US; (2) solid or almost solid lesions (cystic portion <25%); (3) with pathological confirmation by core-needle biopsy or surgery; (4) no history of treatment such as breast surgery, chemotherapy or radiotherapy for breast cancer. 346 patients met the inclusion criteria and were enrolled in the study. Eight patients were excluded because of incomplete imaging data. Finally, 338 women (mean age, 45.0 years ± 14.8; range 16–84 years) with 361 breast lesions (mean diameter, 17.0 mm ± 10.6; range 4–86 mm) comprised the study group. For patients with multiple lesions, those suspicious ones and the largest ones on conventional US were examined. Single lesion was analyzed in 315 patients and 2 lesions in 23 patients. The flowchart for patient selection is shown in Fig. 1.

276 of the 361 breast lesions in the study have been previously reported in a study to explore the diagnostic performance of combined use of elasticity imaging and US BI-RADS^8^, which was different from the purpose of the present study.

### Image Acquisition

All the US and SWS imaging were performed by one of two board certified operators with 3 years’ experience in breast US and 2 years’ experience in breast SWS imaging. According to the breast US examination guideline of the American Institute of Ultrasound in Medicine^11^, a standard US examination including B-mode and color Doppler imaging was firstly performed. Two orthogonal conventional US images were acquired after basic information registration with patients in supine position. When acquiring conventional US images, the ACUSON S3000 US scanner (Siemens Medical Solutions, Mountain View, CA, USA) was used with a 18L6 linear array transducer (Frequency range: 5.5–18 MHz), and sometimes the 9L4 linear array transducer (Frequency: 4–9 MHz) was used when the breast lesion was too large. SWS imaging of VTIQ was thereafter carried out with the same scanner and the 9L4 linear array transducer. The lesion of interest was placed in the center of the screen and the image was optimized. When acquiring the SWS 2D images, patients were asked to hold respiration for 3–5 seconds. The transducer was fixed and kept perpendicular to the skin with slight pressure. The proper pressure that applied to skin was as slight as possible and meanwhile the B-mode images of the breast lesions could be seen clearly. The optimum size of the sampling box was to cover the breast lesions and part of the surrounding tissue. The SW-quality image was firstly obtained, of which the scale of the color map was fixed. The high quality is displayed in green, whereas inferior quality in yellow or red. Then the SW-velocity map was obtained. The color of the SW-velocity map represents the SWS from high (red), intermediate (yellow or green), to low (blue). The adjustment of velocity scale was usually performed to highlight the breast lesion. The scale of SWS ranges from 0.5 to 10 m/s and adjustments of SW-velocity scale would not change the result of SWS measurement. The SW-ROIs (minimal size, 1 × 1 mm) were put on the areas corresponding high quality on SW-quality image whereas the inferior quality areas were avoided. At least seven SWS measurements were performed for each lesion. For lesions with homogeneous distribution of stiffness, the placement of SW-ROI on the SW-velocity map was random. For lesions with heterogeneous stiffness distribution, two SW-ROIs were placed on the highest stiffness area and the lowest stiffness area respectively, and the remaining five SW-ROIs were placed randomly in the lesion, depending on the different colors visualized on SW-velocity map.

### Image Interpretation

**Figure 4:**
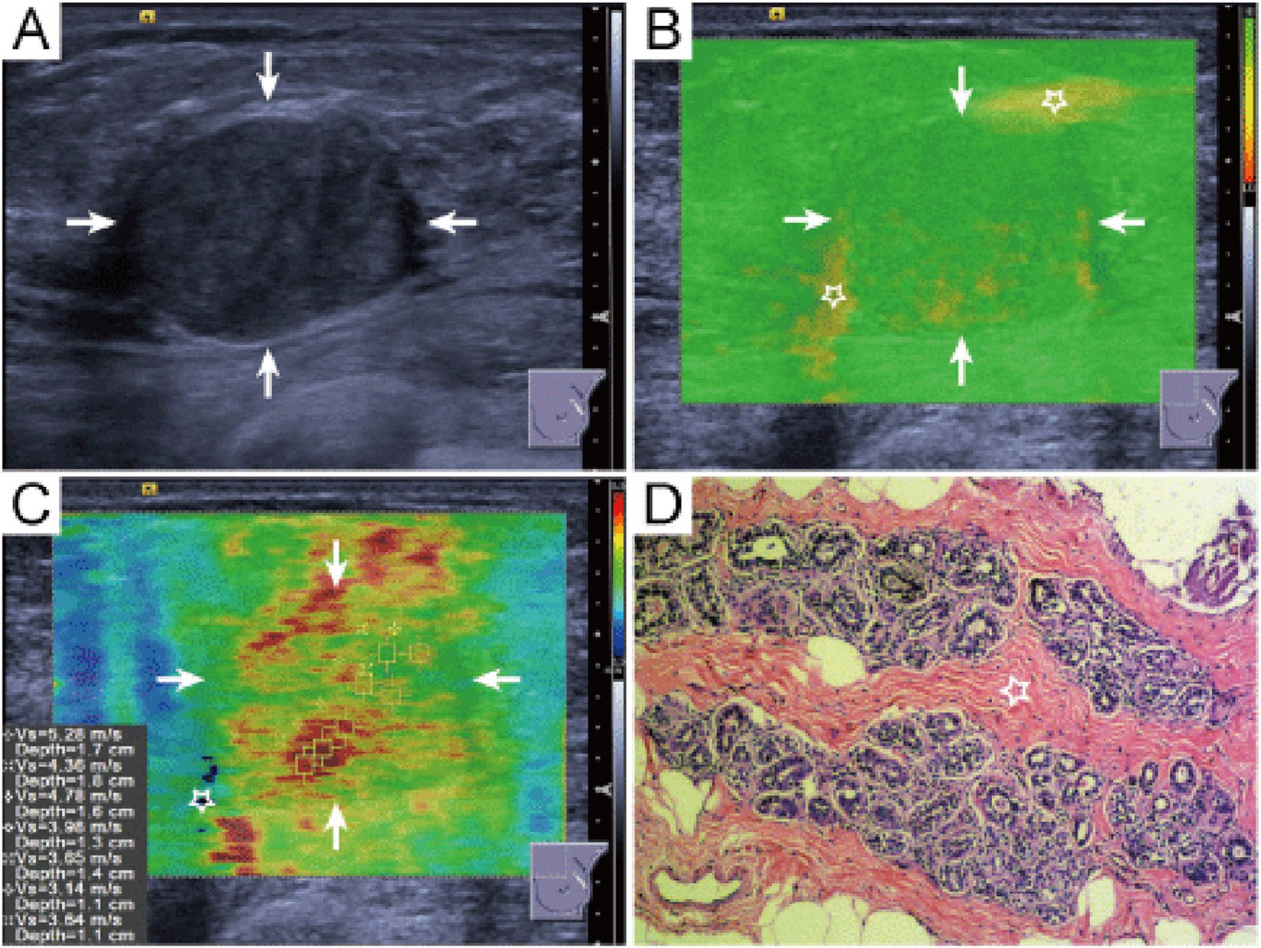
Fibroadenoma in a 35 years old woman. (**A**) The lesion (arrows) is classified as US BI-RADS category 4a on B-mode US. (**B**) The periphery of lesion (arrows) is covered by yellow color (five-pointed star) indicating low QM on QM mode of shear wave speed imaging. (**C**) The SWS_max_ of the lesion (arrows) is 5.28 m/sec. (**D**) Pathological examination confirms the diagnosis of fibroadenoma. The five-pointed star shows the dense fibrillar component. (hematoxylin-eosin stain; original magnification, ×100). QM = quality measurement.

**Figure 5:**
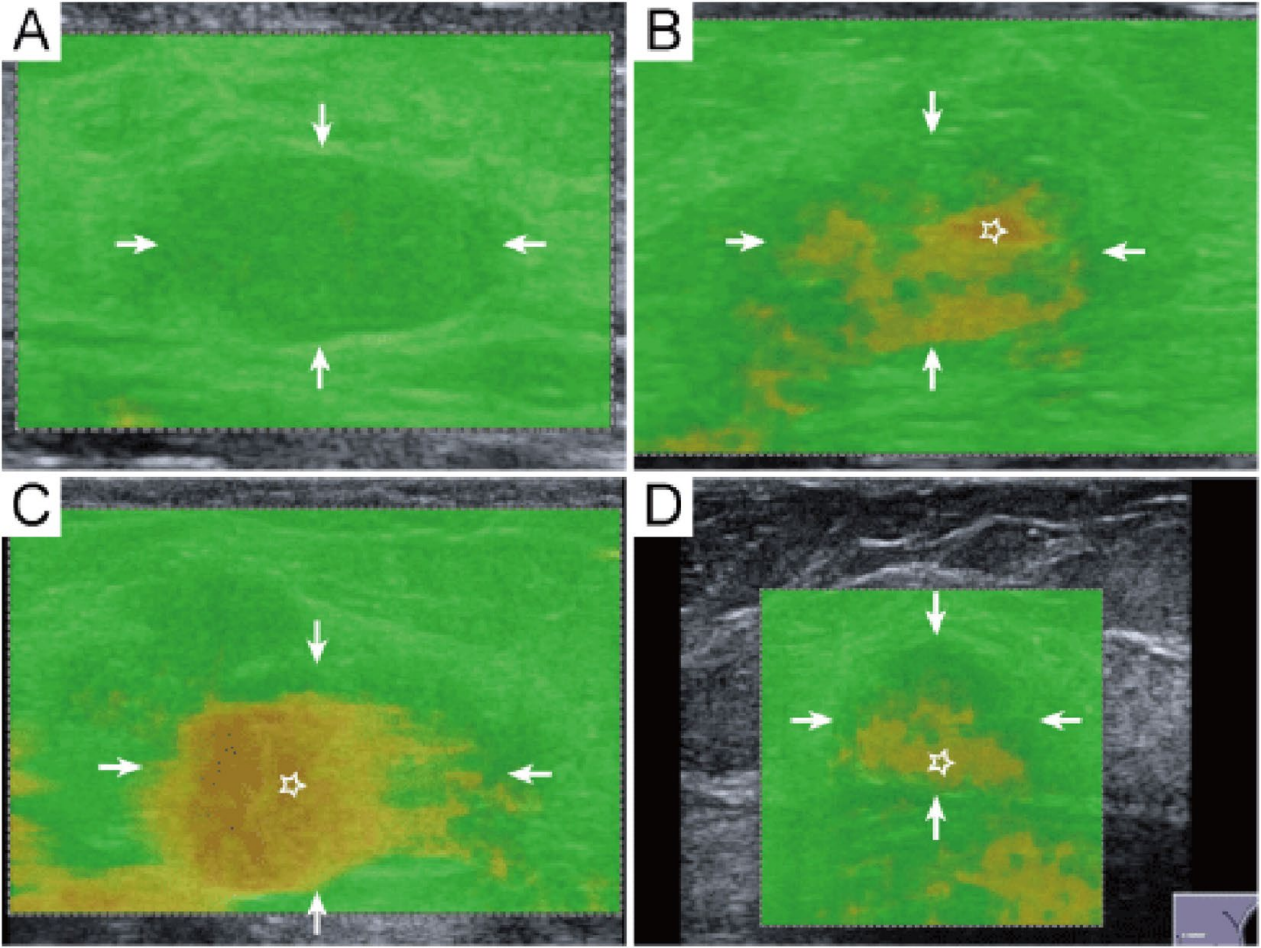
The evaluation standard of QM. (**A**) High QM-the whole breast lesion (arrows) is covered by green color on QM image. (**B**–**D**) Low QM-the lesion or surrounding rim is covered by yellow or red color (five-pointed star). QM = quality measurement.

At the same setting, all the characteristics of conventional US images and QM on SW-quality images of breast lesions were interpreted by another two independent blind observers, who both had more than 3 years’ experience with breast US and more than 2 years’ experience with breast SWS imaging. A third blind observer who had more than 20 years’ experience with breast US and more than 3 years’ experience with breast SWS imaging made the final decision when disagreements were encountered. As to the conventional US images, the following features of the breast lesions were interpreted, including shape (oval/round, irregular), orientation (parallel, not parallel), margin (circumscribed, non-circumscribed), echo pattern (isoechoic, complex cystic and solid, hypoechoic), posterior features (unchanged, changed), calcifications (present, absent), and vascularity (present, absent) on color Doppler images. Changed posterior features included posterior acoustic enhancement, acoustic shadowing and their combination. The interpretation of conventional US images for breast lesions was referred to the latest US BI-RADS of American College of Radiology (ACR)^12^. In the 2D SW-quality image, if the lesion and surrounding rim appeared yellow or red color, the QM of breast lesions was regarded as low^9^ (Fig. 4). On SW-velocity image, the areas corresponding low QM are often displayed in black, namely “no signal detected”. On the other hand, if the whole breast lesion was covered by green color on SW-quality image, the QM was regarded as high (Fig. 5). In the SW-velocity mode, the SWS_*max*_ values of breast lesions were used in the analysis.

### Data Analysis

The possible associated factors for QM on SWS imaging were explored using binary logistic regression, which included patient age, maximum lesion depth (i.e. from skin surface to lesion bottom), lesion diameter, pathological diagnosis, shape, orientation, margin, echo pattern, posterior feature, calcification, intralesional blood flow and SWS_*max*_ of lesions.

In addition, according to classification criteria of QM described above, all breast lesions were divided into two groups (low QM and high QM). The diagnostic performance for breast lesions was evaluated thereafter for three methods including SWS_*max*_ alone, QM alone and SWS_*max*_+QM. Using SWS_*max*_ alone, breast lesions with SWS_*max*_ greater than or equal to the cut-off SWS_*max*_ value were considered as malignancy whereas the remaining breast lesions were considered as benign. In the method of QM, breast lesions with low QM were considered as malignant and breast lesions with high QM were considered as benign. In the method of SWS_*max*_+QM, breast lesions with SWS_*max*_ greater than or equal to the cut-off value were still considered as malignancy. In addition, those with both low SWS_*max*_ (<cut-off value) and low QM were also considered as malignancies. And the remaining lesions were considered as benign. Subgroup analysis was also performed according to diameters of the lesions (Group1, ≤10 mm; Group 2, 11–20 mm; Group 3, >20 mm).

### Inter-operator and intra-operator consistency

According to the method of SWS imaging described above, another 30 breast lesions were tested to evaluate the inter-operator and intra-operator consistency for SWS measurement and QM evaluation. For the inter-operator consistency, SWS measurement and QM evaluation was independently performed by two operators in the same day. Regarding to the intra-observer consistency, repeated SWS measurement and QM evaluation of the same lesion were performed by the same operator in two different days. All the 30 breast lesions were not included into the final analysis.

### Statistical Analysis

All statistical analyses were conducted using the SPSS software (version 20.0; SPSS, Chicago, III) and the SAS version 9.2.1 software (SAS Inc., Cary, NC, USA). The mean values of continuous data were expressed as mean ± standard deviation when they fitted normal distribution and the difference was compared using independent *t* test. The categorical variables were compared using *χ2* test or Fisher’s exact probability test. Receiver operating characteristic (ROC) curve was plotted on SWS, QM and SWS_*max*_+QM to determine cut-off values for them. The indexes of diagnostic performance for the three methods described above including sensitivity, specificity, negative predictive value (NPV), positive predictive value (PPV), and area under curve (AUC) were calculated. The comparisons of sensitivities and specificities were performed by McNemar test. After performing multicollinearity diagnosis for logistic regression using Proc Reg, binary logistic regression with a backward stepwise selection method was used to identify associated factors for QM on SWS imaging. Inter-operator and intra-operator consistency of SWS measurement were evaluated by intraclass correlation coefficient (ICC). Inter-operator and intra-operator consistency for QM were evaluated by Kappa value. The null hypothesis was rejected at an α level of 5% (A two-tailed *P* < 0.05).

## Electronic supplementary material

Supplymentary information
