# Supplementary material for: Quality Measurement of Two-dimensional Shear Wave Speed Imaging for Breast Lesions: the Associated Factors and the Impact to Diagnostic Performance
Source: Sci Rep. 2017 Jul 11;7:5076. doi: 10.1038/s41598-017-05281-5 (PMC5506023; doi:10.1038/s41598-017-05281-5)
Supplement: Supplementary file 1 — Supplymentary information [file 41598_2017_5281_MOESM1_ESM.pdf]

Quality Measurement of Two-dimensional Shear Wave Speed Imaging for Breast Lesions: the Associated Factors and the Impact to Diagnostic Performance

(Original research)

Dan-Dan Li, Hui-Xiong Xu*, Bo-Ji Liu, Xiao-Wan Bo, Xiao-Long Li, Rong Wu

Department of Medical Ultrasound, Shanghai Tenth People's Hospital, Ultrasound Research and Education Institute, Tongji University School of Medicine, Shanghai 200072, China. *Correspondence and requests for materials should be addressed to H.X.X (email:xuhuixiong@126.com; Tel: +86-21-66307539; Fax: +86-21-66307539)

Appendix 1 Binary Logistic Regression Analysis in the Prediction of Low Quality in Breast Lesions for Two Independent Observers.

Factors	OR	95% CI	*P*-Value
**Observer 1**			
**Overall (n=361)**			
**Maximum depth**	1.384	1.285, 1.491	< .001
**Calcifications**	2.439	1.157, 5.142	.019
**≤ 10mm Lesions (n=91)**			
**Maximum depth**	1.890	1.327, 2.692	< .001
**Posterior features**	2.071	1.144, 3.748	.016
**11-20 mm Lesions (n=184)**			
**Maximum depth**	1.612	1.397, 1.859	< .001
**Calcifications**	9.124	2.671, 31.170	< .001
**> 20mm Lesions (n=86)**			
**Maximum depth**	1.211	1.097, 1.337	< .001
**Observer 2**			
**Overall (n=361)**			
**Maximum depth**	1.450	1.334, 1.576	< .001
**Posterior features**	1.189	1.025, 1.379	.022
**≤ 10mm Lesions (n=91)**			
**Maximum depth**	2.179	1.324, 3.585	.002
**Margin**	32.366	1.799, 582.458	.018
**11-20 mm Lesions (n=184)**			
**Maximum depth**	1.682	1.452, 1.949	< .001
**Calcifications**	4.806	1.465, 15.762	.010
**> 20mm Lesions (n=86)**			
**Maximum depth**	1.220	1.105, 1.347	< .001

Appendix 2 The Diagnostic Performances of Maximum Shear Wave Speed plus Quality Measurement method for Two Independent Observers.

Group	Cut-off	SEN (95%CI)	*P*-Value	SPE (95%CI)	*P*-Value	NPV (95%CI)	PPV (95%CI)	AUC	*P*-Value
***SWS**$_{max}$*	5.80 m/s	67.3%* (57.1%-76.5%)	< .05*	90.5%* (86.7%-94.1%)	< .001*	88.1% (63.0%-82.1%)	72.5% (83.7%-91.8%)	0.849 (0.808-0.885)	< .05*
**Group1 (n=91)**	3.97 m/s	66.7% (34.9%-90.1%)		72.2% (60.9%-81.7%)		26.7% (12.3%-45.9%)	93.4% (84.1%-98.2%)	0.743 (0.641-0.829)	> .05
**Group2 (n=184)**	6.19 m/s	47.62 (32.0%-63.6%)		95.5% (90.9%-98.2%)		74.1% (53.3%-89.1%)	87.0% (81.0%-91.7%)	0.783 (0.719-0.839)	< .05*
**Group3 (n=86)**	4.99 m/s	90.9% (78.3%-97.5%)		92.9% (80.5%-98.5%)		93% (80.7%-98.6%)	90.7% (77.9%-97.4%)	0.935 (0.861-0.977)	< .05*
**Observer 1**									
**QM alone**	low	65.3% (55.0%-74.6%)	.868	72.2% (66.4%-77.6%)	< .001[$]	84.8% (79.4%-89.3%)	46.7% (38.1%-55.4%)	0.688 (0.637-0.735)	< .001[$]
**Group1 (n=91)**	low	33.3% (9.9%-65.1%)		86.1% (76.5%-92.8%)		89.5% (80.3%-95.3%)	26.7% (7.3%-56.2%)	0.597 (0.489-0.699)	.187[$]
**Group2 (n=184)**	low	66.7% (50.5%-80.4%)		71.1% (62.9%-78.4%)		87.8% (80.4%-93.2%)	40.6% (28.8%-53.2%)	0.689 (0.617-0.755)	.037[$]
**Group3 (n=86)**	low	72.7% (57.2%-85.0%)		50.0% (34.2%-65.8%)		63.6% (45.1%-79.6%)	60.4% (46.0%-73.5%)	0.614 (0.502-0.717)	< .001[$]
***SWS**$_{max}$* **+QM**	—	84.7% (76.0%-91.2%)	< .001[#]	65.4% (59.3%-71.1%)	< .001[#]	92.0% (87.1%-95.4%)	47.4% (40.1%-55.4%)	0.750 (0.702-0.794)	.003[#]
**Group1 (n=91)**	—	58.3% (27.7%-84.8%)		79.8% (69.2%-88.0%)		92.6% (83.7%-97.6%)	30.4% (12.9%-53.5)	0.690 (0.585-0.783)	.162
**Group2 (n=184)**	—	93.8% (82.8%-98.7%)		46.2% (32.2%-60.5%)		88.9% (70.8%-97.6%)	61.6% (49.4%-72.9%)	0.700 (0.600-0.787)	.326
**Group3 (n=86)**	—	95.5% (84.5%-99.4%)		47.6% (32.0%-63.6%)		90.9% (70.2%-99.0%)	65.6% (52.7%-77.1%)	0.715 (0.608-0.808)	.003[#]
**Observer 2**									
**QM alone**	low	63.3% (52.9%-72.8%)	.608	75.3% (69.6%-80.4%)	< .001[$]	84.6% (79.3%-89.0%)	48.8% (39.9%-57.8%)	0.693 (0.642-0.740)	< .001[$]
**Group1 (n=91)**	low	25.0% (5.5%-57.2%)		91.1% (82.6%-96.4%)		88.9% (80.0%-94.8%)	30.0% (6.7%-65.2%)	0.581 (0.473-0.683)	.080
**Group2 (n=184)**	low	61.9% (45.6%-76.4%)		72.5% (64.4%-79.7%)		86.6% (79.1%-92.1%)	40.0% (27.9%-53.0%)	0.672 (0.599-0.739)	.016[$]
**Group3 (n=86)**	low	75.0% (59.7%-86.8%)		54.8% (38.7%-70.2%)		67.6% (49.5%-82.6%)	63.5% (48.8%-76.5%)	0.649 (0.538-0.749)	< .001[$]
***SWS**$_{max}$* **+QM**	—	82.7% (73.7%-89.6%)	< .001[#]	66.9% (60.9%-72.6%)	< .001[#]	91.2% (86.3%-94.8%)	48.2% (40.5%-56.0%)	0.661 (0.555-0.757)	.011[#]
**Group1 (n=91)**	—	50.0% (21.1%-78.9%)		82.3% (72.1%-90.0%)		91.5% (82.5%-96.8%)	30.0% (11.5%-55.0%)	0.649 (0.542-0.746)	.230
**Group2 (n=184)**	—	93.8% (82.8%-98.7%)		48.1% (34.0%-62.4%)		89.3% (71.8%-97.7%)	62.5% (50.2%-73.7%)	0.709 (0.610-0.796)	.234
**Group3 (n=86)**	—	95.5% (84.5%-99.4%)		50.0% (34.2%-65.8%)		91.3% (71.4%-99.0%)	66.7% (53.7%-78.0%)	0.727 (0.621-0.818)	.025[#]

Group1, ≤ 10 mm; Group 2, 11 - 20 mm; Group 3, > 20 mm

95%CI=95% confidence interval

AUC= the area under the curve;

QM=quality measurement;

SWS_{max}= maximum shear wave speed;

NPV= negative predictive value;

PPV= positive predictive value.

SEN= sensitivity;

SPE= specificity;

* indicates a significant difference SWS_{max} vs. SWS_{max} + QM

# indicate a significant difference QM vs. SWS_{max} + QM

$ indicate a significant difference QM vs. SWS_{max}
